# Genome-Wide Characterization of the Maize (*Zea mays* L.) WRKY Transcription Factor Family and Their Responses to *Ustilago maydis*

**DOI:** 10.3390/ijms241914916

**Published:** 2023-10-05

**Authors:** Yang Wang, Wangshu Li, Jianzhou Qu, Fenghai Li, Wanli Du, Jianfeng Weng

**Affiliations:** 1 Specialty Corn Institute, College of Agronomy, Shenyang Agricultural University, Dongling Street, Shenhe District, Shenyang 110866, China; wy21062107@163.com (Y.W.); qujz0220@syau.edu.cn (J.Q.); lifenghai@126.com (F.L.); 2 Institute of Crop Science, Chinese Academy of Agricultural Sciences, Zhongguancun South Street, Haidian District, Beijing 100081, China; liwangshu1@126.com

**Keywords:** maize, WRKY transcription factor, genome-wide identification, *Ustilago maydis*

## Abstract

Members of the WRKY transcription factor (TF) family are unique to plants and serve as important regulators of diverse physiological processes, including the ability of plants to manage biotic and abiotic stressors. However, the functions of specific WRKY family members in the context of maize responses to fungal pathogens remain poorly understood, particularly in response to *Ustilago maydis* (DC.) Corda (*U. maydis*), which is responsible for the devastating disease known as corn smut. A systematic bioinformatic approach was herein employed for the characterization of the maize WRKY TF family, leading to the identification of 120 *ZmWRKY* genes encoded on 10 chromosomes. Further structural and phylogenetic analyses of these TFs enabled their classification into seven different subgroups. Segmental duplication was established as a major driver of *ZmWRKY* family expansion in gene duplication analyses, while the Ka/Ks ratio suggested that these *ZmWRKY* genes had experienced strong purifying selection. When the transcriptional responses of these genes to pathogen inoculation were evaluated, seven *U. maydis-*inducible *ZmWRKY* genes were identified, as validated using a quantitative real-time PCR approach. All seven of these WKRY proteins were subsequently tested using a yeast one-hybrid assay approach, which revealed their ability to directly bind the *ZmSWEET4b* W-box element, thereby controlling the *U. maydis*-inducible upregulation of *ZmSWEET4b*. These results suggest that these WRKY TFs can control sugar transport in the context of fungal infection. Overall, these data offer novel insight into the evolution, transcriptional regulation, and functional characteristics of the maize WRKY family, providing a basis for future research aimed at exploring the mechanisms through which these TFs control host plant responses to common smut and other fungal pathogens.

## 1. Introduction

As an essential global cereal crop, maize (*Zea mays* L.) is widely used as a source of food, feedstock, industrial raw materials, and energy throughout the world. The cultivation of maize, however, can be hampered by a range of persistent biotic stressors, with diseases caused by fungal pathogens representing one of the most important threats to global corn production. Common smut caused by *Ustilago maydis* (DC.) Corda (*U. maydis*) is among the most deleterious fungal diseases capable of impacting maize crops, resulting in the infection of the aerial portions of maize plants and the consequent growth of tumor-like galls within susceptible tissues [1]. Upon infection, fungal hyphae can grow intracellularly within host plants, traversing cells and establishing a complex interactive network that limits nutrient availability and suppresses the induction of host defense responses [2,3]. Owing to its widespread nature and the ease with which it can be transmitted, common smut represents a major threat to maize production in China and throughout the globe [4]. As different pathogens infect their target hosts using a variety of strategies, plants have evolved a complex immune system comprised of a series of tightly connected signal transduction networks regulated by specific transcription factors (TFs), including members of the WRKY, bHLH, NAC, AP2/EF, and TGA/bZIP families. WRKY TFs, in particular, have been established as vital mediators of early transcriptional responses in plants necessary for appropriate immune response induction [5]. The research focused on elucidating the mechanisms through which these WRKY family TFs function is thus crucial as a means of providing greater insight into the processes that shape fungal resistance. Members of the WRKY TF family, which are unique to plants, harbor a highly conserved N-terminal WRKYGQK region and C-terminal zinc-finger motifs, both of which are essential for the ability of these proteins to bind to target DNA sequences [6,7]. WRKY TFs function by binding to specific W-box elements located in target gene promoter regions, thereby inducing or suppressing transcriptional activity [8]. The numbers of WRKY domains and the zinc finger motif patterns in WRKY family TFs can be used to classify them into three groups. These include Group I proteins that generally harbor both N- and C-terminal WRKY domains as well as a C_2_H_2_ zinc finger motif, Group II proteins with one WRKY domain followed by a C_2_H_2_-type zinc finger motif that in further stratified into the IIa, IIb, IIc, IId, and IIe subgroups in phylogenetic analyses, and Group III proteins with a single WRKY domain and a distinct C_2_H_C_-type motif [9,10]. In recent decades, a growing body of research has elucidated the functional characteristics of specific WRKY TFs in plants, demonstrating that they play important roles in the regulation of JA and SA-mediated disease resistance [11,12], heavy metal tolerance [13], tolerance to salinity and drought [14,15], cold tolerance [16], and the biosynthesis of secondary metabolites [17]. Few studies to date, however, have focused on the functional roles of WRKY family TFs as regulators of maize fungal disease resistance.

Glycogen, together with fructose, glucose, xylose, and galactose, is transported by various types of sugar transporters across the cell membrane to provide energy for the organism, thus playing a crucial role in plant growth and development. The Sugars Will Eventually be Exported Transporters (SWEETs) are involved in several vital processes, where they regulate processes such as plant–pathogen and source–sink interactions, amongst others, in plants [18]. As pathogens compete with plants for nutrients, they have evolved various mechanisms to increase apoplasmic sugar concentrations by exploiting host SWEETs. In rice, increased expression of the *OsSWEET11*, *OsSWEET13*, and *OsSWEET14* genes induced by *Xanthomonas oryzae* (Xoo)enhances sucrose efflux from phloem parenchymal cells to the apoplast, a process that enhances *Xoo* growth and replication [19,20]. In addition to *Xoo*, *Rhizoctonia solani* infection promotes the expression of *OsSWEET11* and *OsSWEET14* in rice leaves [21,22]. Similar to rice, infection of *Arabidopsis* plants by the protist *Plasmodiophora brassicae* leads to the phloem-specific accumulation of *AtSWEET11* and *AtSWEET12* at the site of infection, which facilitates the delivery of sugars to the pathogen [23]. In maize, the biotrophic fungus *U. maydis* promotes massive host cell proliferation and cell expansion, resulting in the formation of tumors. The goal of the fungus is to feed on plant resources, especially sugars and free amino acids [2,24]. SWEET transcription has been shown to be increased following *U. maydis* invasion, leading to the recruitment of SWEET transporters for leakage of sugars into the apoplasm at the biotrophic interface, while the sugars can also be transported back into the intracellular environment via the maize-*U. maydis* interaction [25]. However, there are few studies focusing on the transcriptional regulation of SWEET genes by WRKY TFs. 

Maize is widely used as a model for genomic and genetic research efforts. Rapid advances in whole-genome sequencing technologies have yielded a more complete and accurate maize reference genome, providing an opportunity for the genome-wide characterization of particular genes. Here, a genome-wide screening strategy was implemented to identify and evaluate the *ZmWRKY* gene family in maize, including analyses of the chromosomal locations, conserved motifs, structures, and evolutionary history. A transcriptomic atlas of changes in the expression of these *ZmWRKY* family genes was also generated in tissues following *U. maydis* inoculation. Yeast one-hybrid (Y1H) was used as an approach to assess the binding of specific ZmWRKY TF to the target gene promoter, and the qPCR approach was employed to evaluate the effect of the infection on the transcription of specific TFs. Together, these results will offer an important foundation for future efforts aimed at the more reliable breeding of maize varieties that are more resistant to common smut. 

## 2. Results

### 2.1. WRKY Genes Play Important Regulatory Roles in Response to U. maydis Inoculation

**Figure 1:**
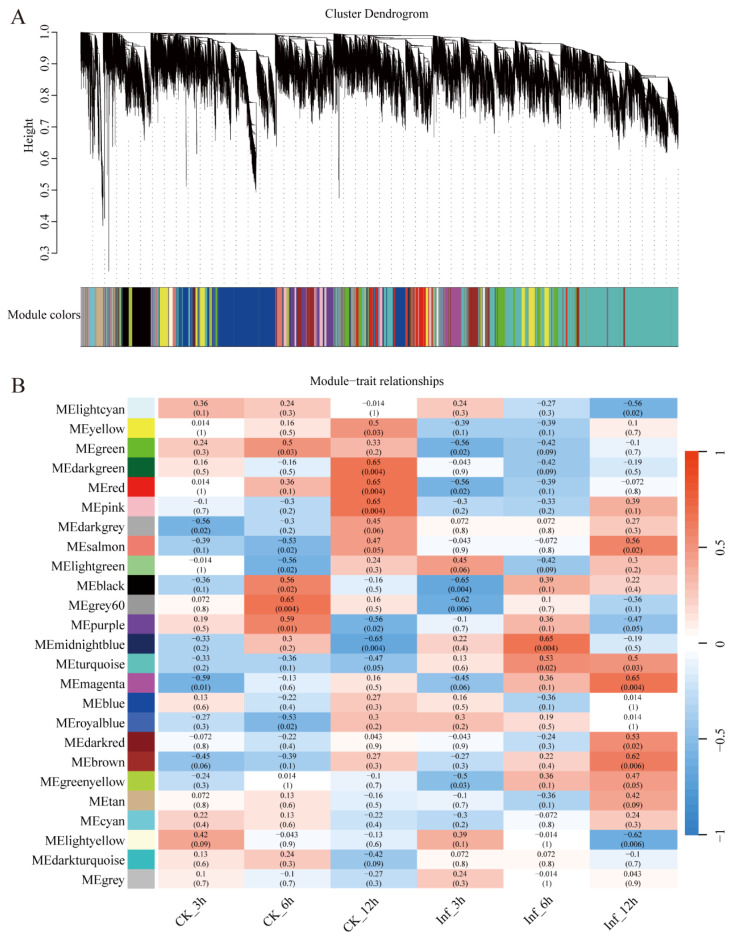
Weighted gene co-expression network analysis. (**A**) A hierarchical clustering tree of the established modules. Each branch in the clustering tree represents a gene, while height in the vertical axis is the clustering distance. (**B**) A heat map representing correlations between modules and treatments.

To explore the potential mechanistic basis for maize defense responses against *U. maydis*, RNA sequencing analyses of leaf tissues harvested at 3, 6, and 12 h post-infection were collected, with distilled water serving as a control (CK). In a prior report, comparisons of these gene expression profiles revealed 7,996 differentially expressed genes (DEGs) [26]. To expand on these results and to clarify the gene clusters most closely related to *U. maydis* infection, a WGCNA was performed using maize leaf samples (CK or 3, 6, or 12 h post-infection [hpi]). A systematic clustering dendrogram and a heatmap were used to identify signature gene sets, revealing 25 modules (Figure 1A,B). Filaments and appressoria are reportedly first detectable during *U. maydis* infection at 6 hpi [26]. Correlations between module eigengenes and traits were thus assessed at the 6 hpi time point in this dataset, revealing that of these 25 modules, the MEmidlightblue and MEturquoise modules were significantly correlated with the 6 hpi data (r^2^ = 0.65 and r^2^ = 0.53, respectively). These two modules were thus screened for potential *U. maydis* infection-related genes. In total, 416 TFs were identified, the greatest number of which were WRKY family TFs (*n* = 47), followed by members of the ERF family (*n* = 45) and the NAC and MYB family (*n* = 37) (Table S1). As the members of the *ZmWRKY* gene family were more abundant than other TFs in this co-expression network conducted following *U. maydis* infection, this suggests that research focused on this TF family may offer new mechanistic insights into how maize plants resist or respond to common smut.

### 2.2. ZmWRKY Gene Identification and Characterization

**Figure 2:**
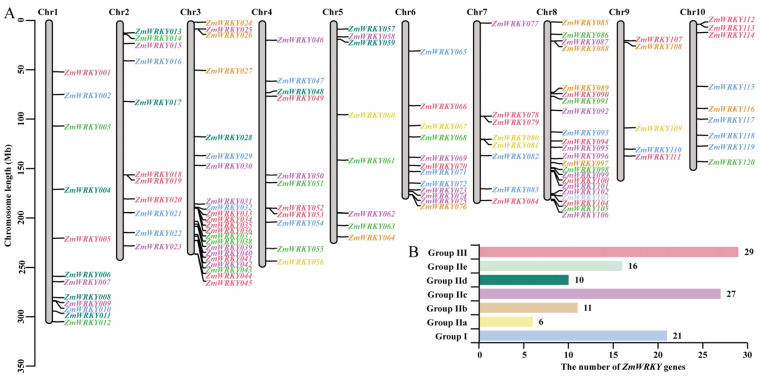
*ZmWRKY* gene chromosomal locations. (**A**) The genomic locations of *ZmWRKY* genes in ten chromosomes. (**B**) Numbers of *ZmWRKY* genes in the indicated groups. Different *WRKY* gene groups are denoted with different colors, including pink represents Group III, light green represents Group IIe, dark green represents Group IId, purple represents Group IIc, dark yellow represents Group IIb, light yellow represents Group IIa, blue represents Group I.

Next, the Zm-B73-REFERENCE-NAM-5.0 (RefGen_v5) reference genome was leveraged to systematically evaluate and characterize the *ZmWRKY* gene family. In total, 120 *ZmWRKY* genes distributed across 10 chromosomes were detected and numbered according to their chromosomal locations (*ZmWRKY1-120*) (Table S2, Figure 2). The encoded ZmWRKY proteins were predicted to vary in length from 125 amino acids (ZmWRKY117) to 729 amino acids (ZmWRKY054), with predicted molecular weights from 14.03 (ZmWRKY117) to 78.73 (ZmWRKY054) kDa, and isoelectrc point values ranging from 4.88 (ZmWRKY105) to 10.12 (ZmWRKY013) (Table S2).

### 2.3. Phylogenetic and Conserved Motif Analyses of ZmWRKY Family Genes

**Figure 3:**
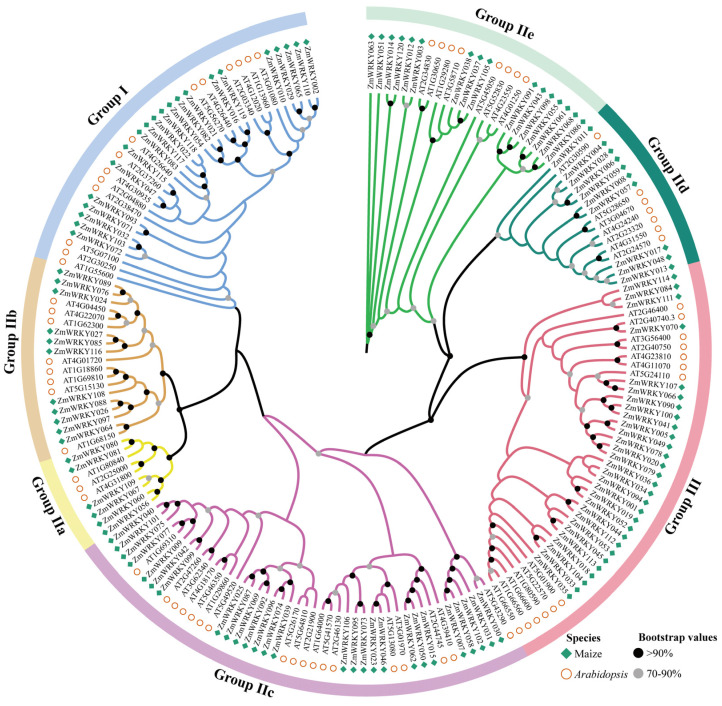
Phylogenetic analyses of maize and Arabidopsis *WRKY* genes. Different *WRKY* gene groups are denoted with different colors, including pink represents Group III, light green represents Group IIe, dark green represents Group IId, purple represents Group IIc, dark yellow represents Group IIb, light yellow represents Group IIa, blue represents Group I.

Phylogenetic relationships among these *ZmWRKY* genes were evaluated by constructing a neighbor-joining tree based on 71 published *Arabidopsis thaliana* WRKY (AtWRKY) proteins (https://www.arabidopsis.org/) and these 120 ZmWRKY proteins. In the resultant tree, these WRKY proteins were classified into three distinct groups (Groups I, II, and III) containing 35, 114, and 42 proteins, respectively. Proteins in Group II were further subclassified into the II-a, II-b, II-c, II-d, and II-e subgroups containing 9, 19, 45, 17, and 24 members, respectively (Figure 3).

**Figure 4:**
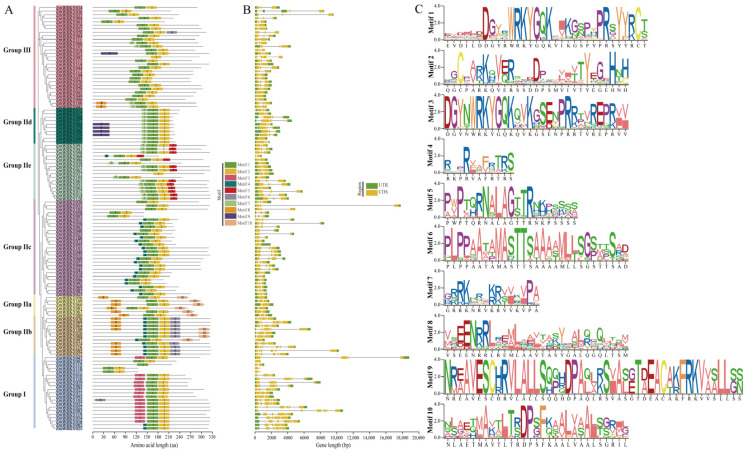
ZmWRKY conserved motif and gene structure analyses. (**A**) ZmWRKY protein motif composition. Protein motif types are shown to the right, with 10 total MEME motifs being shown in individual colors. (**B**) ZmWRKY gene structures. The untranslated regions (UTRs) and coding sequence (CDS) are marked with green and yellow boxes, respectively. The scale for motif and gene structure analyses is shown at the bottom of the panel. (**C**) The MEME motif sequence logos for the 10 motifs identified in ZmWRKY proteins. In total, 10 distinct MEME-motifs are depicted in different colors, and their sequences are listed in Table S3. The length of relevant gene structures and motif components can be estimated with respective scales at panel bottom.

To better clarify the structural characteristics of *ZmWRKY* family genes, the exon-intron structures and the presence of conserved motifs within these genes were evaluated, and phylogenetic clustering was again performed. In total, 21 ZmWRKY family proteins were classified into Group I, including 15 proteins harboring two intact WRKY motifs and C_2_H_2_-type zinc finger motifs, while the remaining members of Group I (ZmWRKY02, ZmWRKY21, ZmWRKY82, ZmWRKY115, ZmWRKY117, and ZmWRKY118) each harbored only a single complete WRKY motif. Group II included 70 ZmWRKY proteins containing a single WRKY motif and a C_2_H_2_-type zinc finger structure. Group III included 29 ZmWRKY proteins with a single WRKY domain that were classified based on their C_2_HC zinc-finger structure. Group II was also further subdivided into Group IIa (6), Group IIb (11), Group IIc (27), Group IId (10), and Group IIe (16) (Figure 4A). Gene structure analyses revealed variations in the numbers of exons per gene, which ranged from 1–6, 1–6, and 1–4 in Groups I, II, and III, respectively (Figure 4B).

MEME was additionally used to evaluate the conserved domains present within proteins encoded by these *ZmWRKY* family genes, leading to the identification of 10 conserved motifs. Of these, Motifs 1 and 2 were the most highly conserved across these ZmWRKYs. Similar motif compositions were also generally observed within a given group, as in the case of proteins in Groups II-a and II-b, which harbored Motifs 8 and 10. Other motifs were group-specific, including Motif 3 (Group I) and Motif 5 (Group II-e). Motifs, 4, 6, and 9 were found not to co-exist within any single ZmWRKY protein (Figure 4C). This may be a consequence of the evolutionary history of these *ZmWRKY* gene members. Together, these data clearly showed the diversification of the maize ZmWRKY protein family.

### 2.4. Synteny Analyses of the ZmWRKY Gene Family

**Figure 5:**
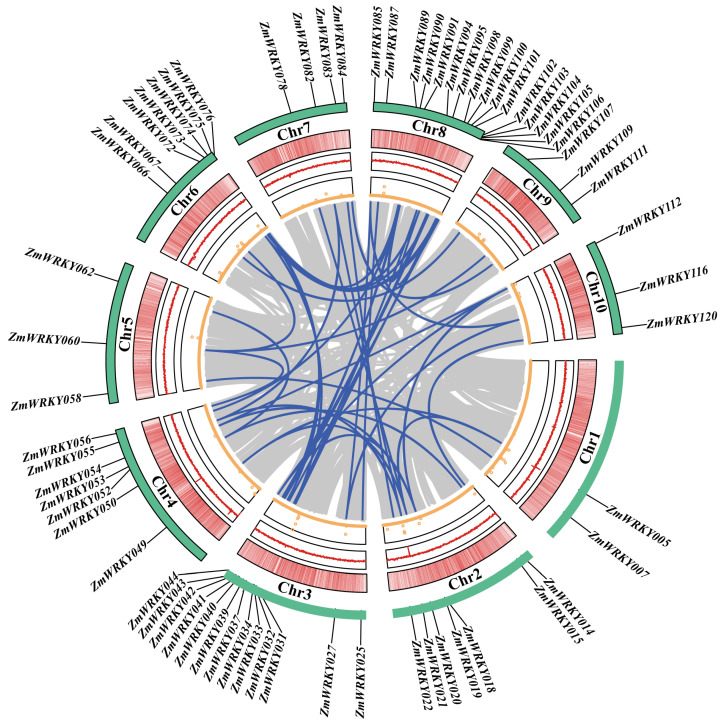
Genome-wide syntenic gene pair identification. Background genome-wide collinear blocks are presented in gray and duplicated *ZmWRKY* gene pairs are linked by connecting red curves. In addition, point plot for N-ratio distribution and line plot for GCskew and each maize chromosome were attached with 300-kb gene density information depicted by heatmap.

Gene duplication is a major driver of genomic evolution. As such, the whole maize genome was evaluated in an effort to gain insight into the evolution of the *ZmWRKY* gene family. This approach revealed 50 segmentally duplicated gene pairs (Figure 5 and Table S4), whereas no evidence of tandem gene pair duplication events was detected. This suggests that segmental duplication events have served as the major driver of the emergence of new *ZmWRKY* genes. 

**Figure 6:**
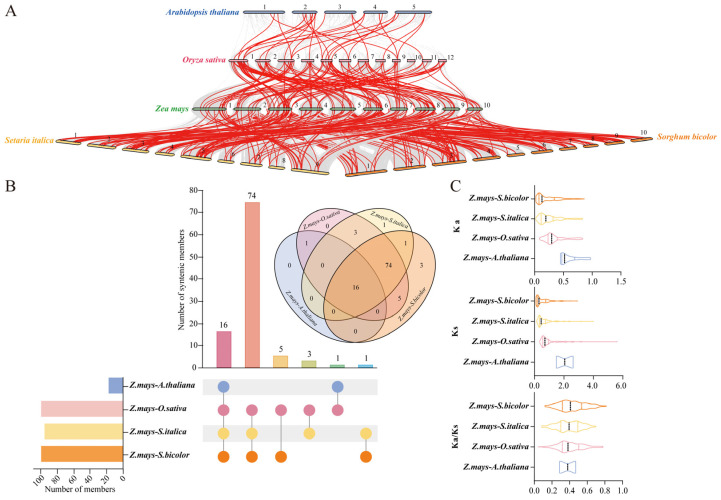
Collinearity and Ka/Ks analyses. (**A**) The collinearity of *ZmWRKY* genes with genes from four representative species was analyzed, with gray representing collinear genes and red highlighting connections between syntenic genes. (**B**) Numbers of homologous genes identified in maize and evaluated representative species. The number of members, which represents syntenic WRKY members, is shown along the vertical axis, while the number of syntenic members, which represents non-redundant syntenic WRKY members in maize and other species, is on the horizontal axis. (**C**) Nonsynonymous substitutions rate (Ka), synonymous substitutions rate (Ks), and the Ka/Ks ratio values were analyzed for *WRKY* genes encoded in maize and representative species, including *Arabidopsis thaliana*, *Oryza sativa*, *Setaria italica*, and *Sorghum bicolor*.

To explore the syntenic relationships between *ZmWRKY* genes and WRKYs encoded by other species, a synteny analysis was performed for four plant species (*Arabidopsis thaliana*, *Oryza sativa*, *Setaria italica*, and *Sorghum bicolor*) (Figure 6A, Figure S1). This analysis revealed 19 orthologous gene pairs in *A. thaliana*, consistent with a distant evolutionary relationship between the two species (Figure 6A, Figure S1, and Table S5). In contrast, 151, 152, and 159 orthologous gene pairs were detected in *O. sativa*, *S. italica*, and *S. bicolor*, respectively, highlighting the closer phylogenetic relationships among these species and indicating that they may have arisen from a shared ancestor (Figure 6A, Figure S1, and Tables S6–S8). Non-redundant *ZmWRKY* genes exhibiting syntenic relationships with these other species were filtered, revealing that 15 of these *ZmWRKYs* were associated with orthologous genes in all analyzed species (Figure 6B). The Ka/Ks ratio for these orthologous *ZmWRKY* gene pairs was less than 1 (Figure 6C), indicating that the members of this gene family have experienced strong purifying selection over the course of evolution.

### 2.5. Analyses of Tissue-Specific ZmWRKY Gene Expression Patterns

**Figure 7:**
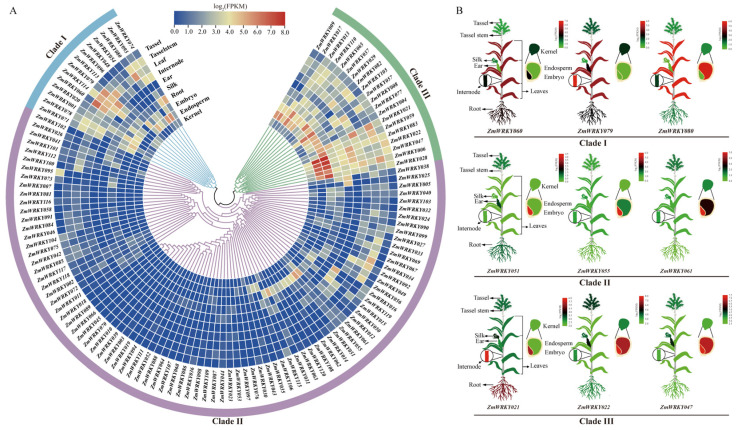
Expression atlas of *ZmWRKY* genes in multiple maize tissue types. (**A**) *ZmWRKY* gene expression profiles in different tissues. Clades are represented in different colors. (**B**) Heatmaps representing the expression of representative *ZmWRKY* genes from Clades I-III. FPKM values were subjected to log_2_ normalization.

To begin evaluating the functional roles of these ZmWRKY family members in the context of maize growth and development, a *ZmWRKY* spatiotemporal expression atlas was prepared using tissue samples including maize kernels collected at 14 days after pollination (DAP), endosperm samples collected at 27 DAP, embryo samples collected at 27 DAP, and root, silk, ear, internode, leaf, tassel stem, and tassel samples (Table S9). In general, the *ZmWRKY* genes in different groups varied with respect to their expression patterns. The majority of Clade I *ZmWRKY*s, for example, were expressed at the highest levels in tassel, stem, leaf, internode, and embryo-27DAP samples, suggesting that they may be important in these tissues. Most Clade II *ZmWRKY*s were expressed at lower levels in all analyzed tissues, potentially suggesting that they interact with other proteins or function synergistically in the context of maize development. Clade III *ZmWRKY*s were highly expressed in most tissues including the endosperm-27DAP, embryo-27DAP, root, internode, leaf, tassel stem, and tassel tissues, indicating that they may serve as particularly important regulators of the growth and development of maize plants (Figure 7A). Nine representative *ZmWRKY* genes from different clades were selected to construct a heatmap in order to provide a visual overview of these expression patterns (Figure 7B). Together, these results highlight the diverse patterns of *ZmWRKY* gene expression in tissues, underscoring the potentially critical and varied roles that they may play in shaping maize growth.

### 2.6. Analyses of U. maydis Infection-Related Changes in ZmWRKY Gene Expression Patterns

**Figure 8:**
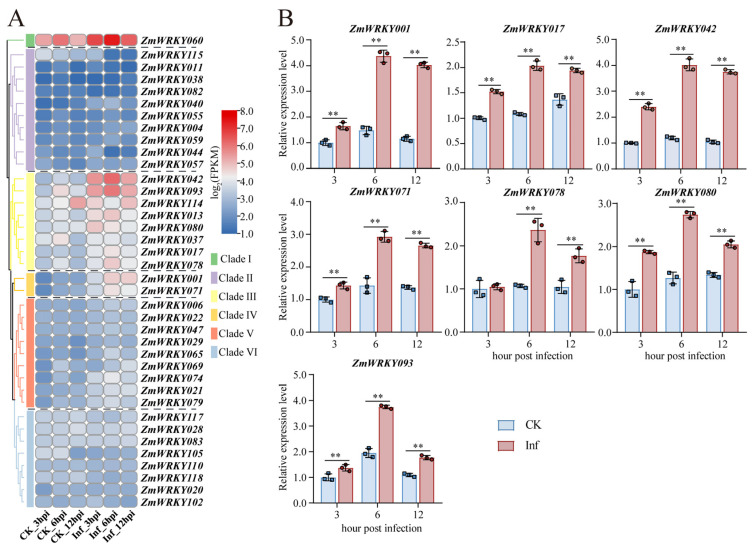
*ZmWRKY* gene expression analysis during *U. maydis* infection. (**A**) *ZmWRKY* gene expression (log_2_ FPKM) over the course of *U. maydis* infection. Clades are indicated with different colors. (**B**) qPCR analyses of seven representative *ZmWRKY* genes from Clades III and IV. Data are means ± standard errors from three technical and biological replicates. ** *p* < 0.01; Student’s *t*-test.

Next, efforts were made to systematically assess the changes in *ZmWRKY* gene expression following *U. maydis* infection by analyzing the expression of these genes at 3, 6, and 12 hpi. In total, 38 *ZmWRKYs* were detected in the control and infected samples with a mean FPKM ≥ 1 across three biological replicates (Table S10). The majority of these *ZmWRKY* genes were expressed at relatively low levels in CK samples, and universally low expression of the *ZmWRKY* genes in Clades II, V, and IV was noted. In contrast, many of the *ZmWRKY* genes in Clade III (e.g., *ZmWRKY017, ZmWRKY042, ZmWRKY078, ZmWRKY080*, and *ZmWRKY093)* and Clade IV (e.g., *ZmWRKY001* and *ZmWRKY071*) were expressed at relatively high levels following *U. maydis* inoculation. Relatively high levels of expression of *ZmWRKY60*, which was assigned to Clade I, were observed in both CK and infected samples (Figure 8A). These diverse patterns of *ZmWRKY* expression may belie the roles that these genes play in the context of maize responses to *U. maydis* infection.

To confirm the RNA-seq results and validate the potential participation of certain *ZmWRKY*s in responses induced by *U. maydis*, *ZmWRKY060* in Clade I (Figure S2); *ZmWRKY038*, *ZmWRKY082*, and *ZmWRKY115* in Clade II (Figure S2); all *ZmWRKY* genes in Clades III and IV (Figure 8B, Figure S2), *ZmWRKY006*, *ZmWRKY021*, and *ZmWRKY022* in Clade V; and *ZmWRKY028*, *ZmWRKY083*, and *ZmWRKY117* in Clade VI (Figure S2) were selected to for qPCR analyses. Not surprisingly, these *ZmWRKY* genes exhibited consistent changes in expression upon *U. maydis* infection compared to those observed in the original transcriptomic dataset. In particular, we refer to the expression level of *ZmWRKY017*, *ZmWRKY042*, *ZmWRKY078*, *ZmWRKY080*, and *ZmWRKY093* in Clade III and *ZmWRKY001* and *ZmWRKY071* in Clade IV, with all seven genes significantly upregulated with the prolongation of the time post-inoculation and more highly expressed in infected samples relative to controls, with the highest induction at 6 hpi (Figure 8B). This suggests a strong correlation between the spatiotemporal dynamics of *ZmWRKY001*, *ZmWRKY017*, *ZmWRKY042*, *ZmWRKY071*, *ZmWRKY078*, *ZmWRKY080*, and *ZmWRKY093* expression and inoculation with *U. maydis*.

### 2.7. Identification of ZmWRKY Proteins Capable of Binding the ZmSWEET4b Promoter

**Figure 9:**
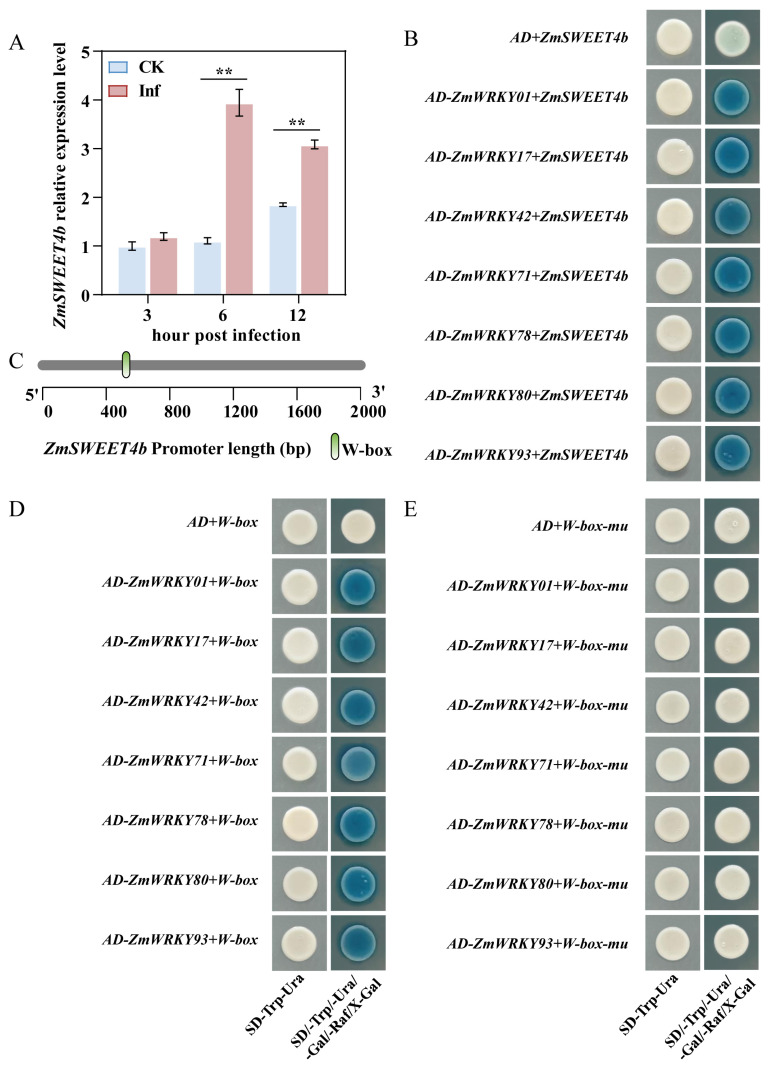
Yeast one-hybrid assay results for analyses of interactions between ZmWRKY proteins and the *ZmSWEET4b* promoter. (**A**) qPCR analyses were used to assess *ZmSWEET4b* expression. Data are means ± standard errors from three technical and biological replicates. ** *p* < 0.01; Student’s *t*-test. (**B**) Identification of ZmWRKY proteins that bind the *ZmSWEET4b* promoter. (**C**) Schematic overview of the W-box element patterns located within 2000 bp upstream of the *-ZmSWEET4b*. (**D**,**E**) Identification of ZmWRKY protein binding to W-box motifs found within the *ZmSWEET4b* promoter. A yeast one-hybrid assay was performed using baits consisting of normal (W-box) (**D**) or mutated (W-box mu) (**E**) three tandem W-box repeats.

Members of the SWEET protein family serve as essential mediators of virulence activity for a range of fungal and bacterial pathogens in *Arabidopsis*, rice, and other species [27,28]. *U. maydis*, for example, can trigger the local upregulation of the sugar transporter gene *ZmSWEET4b*, resulting in the expression of SWEET transporter proteins that can facilitate sugar flow into the apoplast compartment across the biotrophic interface [25]. To test whether ZmWRKY proteins are capable of regulating the expression of *ZmSWEET4b*, an initial qPCR analysis was conducted, which confirmed the profound upregulation of *ZmSWEET4b* following *U. maydis* inoculation. The expression of *ZmSWEET4b* dramatically increased approximately 4 times at 6dpi, 0.5 times more at 12dpi than that of CK after *U. maydis* infection; however, the differences between the two groups were not significant at 3dpi (Figure 9A). A One-hybrid system (Y1H) assay approach was then used to assess the ability of the seven highly expressed ZmWRKYs analyzed above (ZmWRKY001, ZmWRKY017, ZmWRKY042, ZmWRKY071, ZmWRKY078, ZmWRKY080, and ZmWRKY093) to specifically bind the *ZmSWEET4b* promoter. Transformants capable of growing on the SD/-Trp/-Ura medium were obtained for all seven *ZmWRKY* genes, and the co-transformation of yeast with these genes and the *ZmSWEET4b* promoter resulted in the production of a dark blue product upon growth on the SD/-Trp/-Ura/-Gal/-Raf/X-Gal medium, while the same was not evident for the blank control group (Figure 9B). This suggests a marked increase in the expression of the β-galactosidase (LacZ) gene in the experimental groups relative to the control groups, revealing that the *ZmSWEET4b* promoter is subject to self-activation but that the ZmWRKY001/017/042/071/078/080/093 proteins can bind to this promoter and enhance its transcriptional activity.

As WRKY family TFs are capable of specifically recognizing and binding W-box elements within the promoter regions upstream of a given target gene, the *ZmSWEET4b* promoter was evaluated, revealing a W-box element present therein (Figure 9B). To test the specificity of ZmWRKY to W-box binding activity in this context, two sets of fusion vectors were constructed, including one vector harboring three tandem W-box repeats (W-box-pLacZi) and one in which three bases were mutated (W-box-mu-pLacZi) (Table S11). Subsequent analyses revealed that both constructs yielded transformants capable of growing normally on the SD/-Trp/-Ura medium, while yeast strains harboring the pB42AD-ZmWRKY001/017/042/071/078/080/093 and pLacZi-W-box vectors produced blue coloration on the di-deficient X-β-gal substrate (Figure 9D), whereas the same was not true when yeast were instead co-transformed with the pLacZi-W-box-mu vector (Figure 9E). Furthermore, in order to determine the binding ability of ZmWRKY members to the ZmSWEET4b promoter without W-box elements, this promoter region was divided into two parts from 1 to 522 bp (P1) and from 529 to 2000 bp (P2), which were used in the yeast one-hybrid assays (Figure S3A). The transformation products of ZmWRKY001 and *ZmSWEET4b* promoters were used as positive controls, which turned blue when grown on the X-β-gal di-deficient substrate. In contrast, yeast transformed with seven ZmWRKY members and the P1 and P2 constructs were able to grow on the SD-Trp-Ura medium without any corresponding blue coloration on the X-β-gal di-deficient substrate (Figure S3B). The data showed that the ability of these ZmWRKY proteins was specifically bound to W-box elements.

### 2.8. Subcellular Localization Analyses

**Figure 10:**
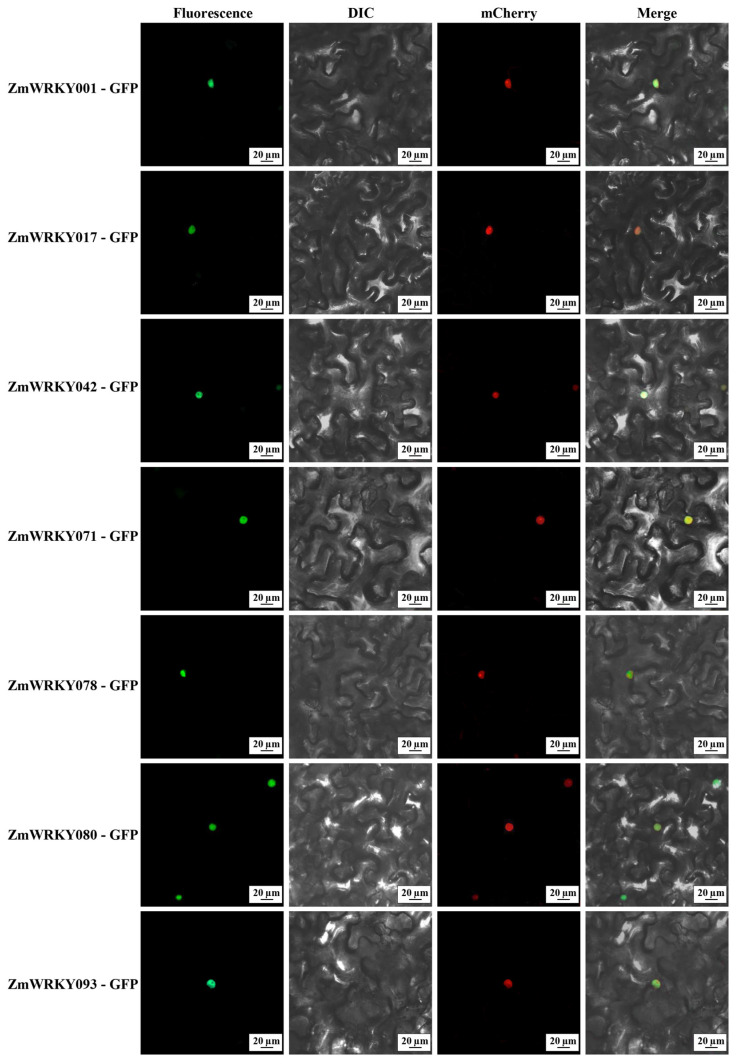
ZmWRKY protein subcellular localization analyses. Scale bar: 20 μm.

The subcellular localization of ZmWRKYs was next investigated. Predictive analyses indicated that 104 of these proteins exhibit nuclear localization, while 6, 5, and 2 localize to the chloroplast, cytosol, and mitochondrial compartments, respectively. In addition, a single ZmWRKY protein was predicted to localize to each of the endoplasmic reticulum, extracellular, and peroxisome compartments. To confirm these predictions, the full-length coding sequences for ZmWRKY001, ZmWRKY017, ZmWRKY042, ZmWRKY071, ZmWRKY078, ZmWRKY080, and ZmWRKY093 were individually fused to the N-terminus of GFP to generate reporter proteins. When expressed in *N. benthamiana* leaves, the fluorescent signal for these ZmWRKYs was exclusively observed in the nucleus (Figure 10), thus confirming the nuclear localization of the ZmWRKY001/017/042/071/078/080/093 proteins.

## 3. Discussion

The plant-specific WRKY TF superfamily has been demonstrated to play diverse regulatory roles in the context of growth, development, and resistance to both biotic and abiotic stressors [29]. The WRKY family exhibits a long evolutionary history that can be traced to the single-celled *Chlamydomonas reinhardtii* green algae, which encodes a single WRKY gene containing two WRKY domain-coding sequences, suggesting that this ancient gene family predates the advent of plant multicellularity [30,31]. Here, the most recent version of the maize reference genome was used to systematically analyze the *ZmWRKY* gene family, revealing 120 non-redundant *ZmWRKY*s encoded within the B73 RefGen_v5 genome (Figure 2 and Table S2). Relative to many other gene families, the WRKY genes exhibit a high degree of evolutionary diversity. The maize genome encodes more *WRKY* genes than barley at 103 [32] or rice at 102 [33], although the wheat genome encodes 124 *WRKY* genes [34]. This may be attributable to the size of the wheat genome, which is 40 and 5 times larger than the rice and maize genomes, respectively, suggesting a positive correlation between genome size and the number of encoded WRKY family TFs. Over the course of its evolution, maize has undergone a minimum of three whole-genome duplication events, contributing to the presence of multiple copies of genes [35,36]. Together, these results suggest that members of the WRKY family have undergone substantial evolutionary expansion in plant species.

Comprehensive analyses of available sequencing data offered insight into WRKY family complexity. Phylogenetic analyses enabled the classification of the 120 identified *ZmWRKY* genes into three major groups based on whether they contained an N-terminal WRKY domain and the characteristics of their C-terminal zinc-finger motifs (C_2_H_2_ or C_2_HC type) (Figure 4). Members of a given phylogenetic group of *ZmWRKY* genes were similar with respect to length, structure, and motif distributions, suggesting that they may play similar roles in cells. These results are consistent with reports from barley, wheat, and common bean genomic analyses [32,34,37]. Certain conserved heptapeptide variations including WKKYGQK (ZmWRKY007), WRKYGEK (e.g., ZmWRKY019, ZmWRKY052, ZmWRKY094), and WRKYGKK (e.g., ZmWRKY037, ZmWRKY069, and ZmWRKY092) were also detected in these analyses, and similar sequences have been reported previously in species including rice and *Arabidopsis* [29,33]. These variable sequences may confer distinct DNA binding specificity or other functional characteristics to these ZmWRKY proteins. In addition, *ZmWRKY115* was found not to contain a whole zinc finger motif, while *ZmWRKY37* and *ZmWRKY45* were found not to contain complete WRKYGQK sequences. The deletion or mutation of these domains may be an important driver of *ZmWRKY* gene family expansion, with the diversification of these characteristic motifs offering a novel basis for future studies of the functions and evolution of these *ZmWRKY* family members.

The evolution of plant genomes is frequently characterized by tandem and segmental duplication events as a mechanism that drives gene amplification and functional diversification [38,39]. Analyses of *ZmWRKY* family gene duplication events revealed a key role for segmental duplication in the evolutionary expansion of this gene family in maize (Figure 5). The uneven distribution of these *ZmWRKY* genes across 10 chromosomes may be attributable to gene duplication events or to the partial fragmental duplication of the maize genome over the course of its evolution [40]. To better understand the evolution of the *ZmWRKY* gene family, synteny analyses of these maize genes and homologous genes in four other plant species were conducted. Relative to the model WRKY gene family in *Arabidopsis*, *ZmWRKYs* exhibited stronger syntenic relationships with the Poaceae family in line with the known evolutionary associations among monocot and eudicot species (Figure 6). This suggests that the syntenic relationships among these *WRKY* genes are associated with the evolutionary divergence of these species. Greater synteny was observed between *Z. mays* and *S. bicolor* relative to that between *Z. mays* and *A. thaliana* (Figure 6), emphasizing their similar genetic relationships and evolutionary history [41]. Of these genes, 15 *ZmWRKY*s were syntenic with the *WRKY* genes in all analyzed species, emphasizing the conservation of these orthologous gene pairs and suggesting that they predate the evolutionary divergence of these species [8]. These intersecting syntenic relationships among *WRKY* genes across species may provide a foundation for future research exploring *WRKY* evolution.

Plants and pathogens are engaged in a perpetual evolutionary arms race such that plants have developed a two-tiered immune response that consists of both pattern- and effector-triggered immunity [42,43]. A growing number of WRKY TFs have been established as key regulators of these two response types. In rice, for example, *OsWRKY53* can reportedly induce oxidative burst activity and inhibit ethylene production to protect against *Nilaparvata lugens* [44]. In *Brassica napus, WRKY15*, *WRKY28*, *WRKY33*, and *WRKY70* have been demonstrated to positively or negatively regulate immune responses [45,46,47,48]. In barley, SnRK1 serves as a metabolic sensor that can promote *WRKY3* degradation and enhance the ability of plants to resist powdery mildew [49]. In rice, the OsMPKK10.2-OsMPK6 signaling axis reportedly facilitates *OsWRKY45* activation and thereby coordinates defenses against *Xanthomonas oryzae* pv. *oryzicola* (*Xoc*) [50,51]. Furthermore, *WRKY3* and *WRKY4* are inducible in response to pathogen infection in *Arabidopsis*, and both the *wrky3* and *wrky4* single and double mutants exhibit more severe disease symptoms and higher levels of fungal growth than wild-type plants after *Botrytis* infection, whereas the overexpression of *WRKY4* greatly enhances plant susceptibility to *P. syringae* [52]. These data provide support for a model wherein specific biological stressors can induce the upregulation of certain WRKY TFs, thereby positively regulating stress-related gene expression such that plants can better tolerate the associated stressors. In the present analysis, patterns of *ZmWRKY* gene expression were evaluated following *U. maydis* infection. A diverse range of *ZmWRKY* expression patterns was noted across clades, indicating that these genes likely play a range of distinct roles in shaping the ability of maize to defend against *U. maydis* (Figure 8A). To better understand the potential relationships between *ZmWRKY* genes and common smut, qPCR analyses were conducted exploring the effects of *U. maydis* infection on seven representative *ZmWRKY* genes. This approach revealed a high degree of *ZmWRKY001*, *ZmWRKY017*, *ZmWRKY042*, *ZmWRKY071*, *ZmWRKY078*, *ZmWRKY080*, and *ZmWRKY093* upregulation following *U. maydis* inoculation, emphasizing the specific activation of these genes in the context of common smut infection.

**Figure 11:**
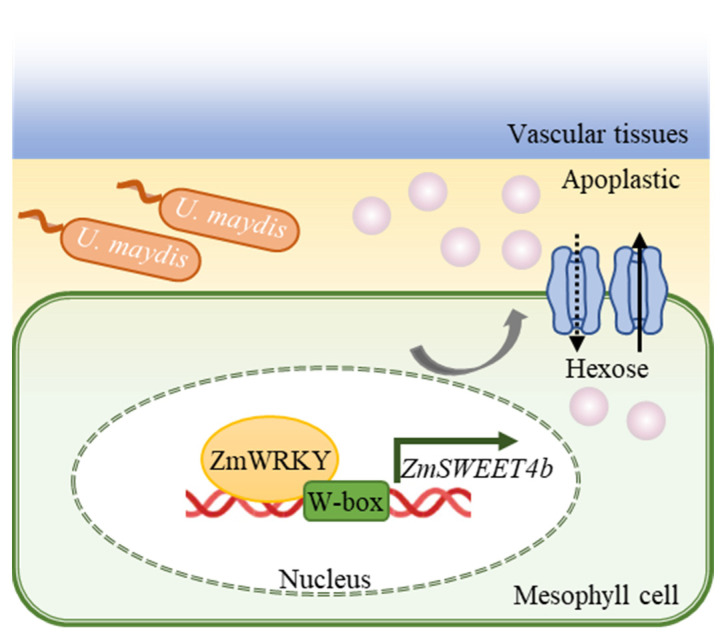
Model overview of the responses to *U. maydis* infection mediated by ZmWRKY proteins. Following *U. maydis* invasion, *ZmWRKY* gene upregulation occurs, and ZmWRKY proteins are able to bind the W-box element in the *ZmSWEET4b* promoter to induce its upregulation, resulting in an increase in sugar efflux from the apoplastic space that is conducive to the growth of *U. maydis*.

Plant immune responses are tightly coordinated by a network of different TFs that govern downstream target gene expression, shaping the ability of plants to resist pathogens and adapt to adverse conditions. WRKY family proteins have been shown to function as transcriptional regulators through their ability to bind to promoter W-box elements upstream of immune-related genes. The expression of the Group I WRKY-induced RBOHB gene, for example, has been demonstrated to induce oxidative burst activity and hypersensitive response (HR)-associated cell death in tobacco plants to protect against *Phytophthora infestans* [53]. WRKY TFs can directly bind the W-box element in the *GhMKK2* promoter and control the transcription of *GhMKK2* induced by *Fusarium oxysporum* f. sp. *vasinfectum* (*Fov*) [54]. Furthermore, the *pCaWRKY40*^−1802 to −1464^ promoter region contains two directly repeated W box-related motifs. Furthermore, the mutation of both W-box motifs significantly blocked *pCaWRKY40* responses to *Ralstonia solanacearum* inoculation (RSI) or heat stress (HS), suggesting that the W-box in *pCaWRKY40*^−1802 to −1464^ is involved in pepper’s responses to RSI and HS [55]. In this study, we proved that the deletion of the W-box element resulted in ZmWRKY001/017/042/071/078/080/093 proteins that bind by default to the P1 and P2 sequences of this promoter (Figure S3). Furthermore, in Figure 9D,E, seven ZmWRKY members bind to the W-box specifically. Thus, these data showed that the ZmWRKY proteins bind to the W-box region upstream of the *ZmSWEET4b* gene, suggesting that these WRKY family proteins activate *ZmSWEET4b* transcription, and the SWEET4b transporter was then recruited to sugars transported into the apoplast (Figure 11).

## 4. Materials and Methods

### 4.1. Plant Materials and U. maydis Inoculation

For this study, an inbred maize variety susceptible to common smut (Ye478) was used in all analyses. The *U. maydis* strain FB1×FB2 pathogen used in these assays was cultured at 28 °C in yeast extract peptone and sucrose light (YEPSL; 0.4% yeast extract, 0.4% peptone, and 2% sucrose) as reported previously [26,56]. Maize seedlings were inoculated with *U. maydis* FB1 × FB2 at three weeks of age, while ddH_2_O was used to treat control plants. Leaves from the same position were harvested from each plant at 3, 6, and 12 h post-inoculation. Three biological replicate samples were collected per time point for downstream analyses.

### 4.2. Genome-Wide Identification of the ZmWRKY TF Family

The ZmWRKY TF family was initially characterized by downloading the maize reference genome (Zm-B73-REFERENCE-NAM-5.0, B73 RefGen_v5) from the Ensembl plant database (http://plants.ensembl.org/index.html, accessed on 8 September 2022), while AtWRKY protein sequences from TAIR (https://www.arabidopsis.org/, accessed on 8 September 2022) were used as queries to identify representative ZmWKY proteins using TBtools (v1.120) (score ≥ 100 and e-value ≤ 1 × 10^−10^) [57]. These ZmWRKYs were then cross-referenced with NCBI BLASTp W (https://blast.ncbi.nlm.nih.gov/Blast.cgi?PROGRAM=blastp&PAGE_TYPE=BlastSearch&LINK_LOC=blasthome, accessed on 8 September 2022). A WRKY domain Hidden Markov Model (HMM) seed file (PF03106) was accessed using the Pfam database (http://pfam.sanger.ac.uk/, accessed on 8 September 2022) and utilized for the identification of ZmWRKY protein with Hmmsearch, scanning all annotated protein sequences in the reference B73 RefGen_v5 genome (e-value 1 × 10^−5^). Non-redundant ZmWRKY protein sequences were then additionally screened with the NCBI Conserved Domain Database (https://www.ncbi.nlm.nih.gov/Structure/cdd/wrpsb.cgi, accessed on 8 September 2022) to confirm the presence of a WRKY domain therein. The molecular weight (MW) and isoelectric point (pI) for each of these ZmWRKYs were predicted using ExPASY (https://web.expasy.org/compute_pi/, accessed on 10 September 2022) [58].

### 4.3. Phylogenetic Classification, Chromosomal Localization, Gene Structure, and Motif Analyses

ClustalW (v1.81) was used for the multiple sequence alignment of *Arabidopsis* and maize WRKY proteins using the default parameters [59]. MEGA 7.0 was then used to construct a neighbor-joining phylogenetic tree with 1000 bootstrap replicates [60]. The classification scheme for AtWRKY proteins was used to assign these ZmWRKYs to distinct groups. The resultant phylogenetic tree was presented using Chiolot (https://www.chiplot.online/, accessed on 10 September 2022) and Adobe Illustrator2020 (24.3.0.569).

The reference genome was used to assess the chromosomal distributions of *ZmWRKY* genes, which were visualized with MapChart (https://www.wur.nl/en/show/Mapchart.html/, accessed on 10 September 2022). The GFF3 annotation file was downloaded from the Ensembl plant database (http://plants.ensembl.org/index.html, accessed on 10 September 2022) after which it was utilized to display the structures of *ZmWRKY* genes in Tbtools (v1.120). MEME (http://alternate.meme-suite.org/tools/meme, accessed on 5 October 2022) was used to conduct a conserved motif analysis of ZmWRKY proteins, identifying up to 10 conserved motifs. MEME-derived motif sequence logos were presented using TBtools (v1.120).

### 4.4. Duplication and Synteny Analyses of ZmWRKY Gene Family

Tandem and segmental duplication events for *ZmWRKY* genes were identified using MCScanX, with the results being presented using TBtools (v.1.120). Synteny analyses assessing the relationships among *WRKY* genes encoded by *Zea mays* and *Arabidopsis thaliana*, *Oryza sativa*, *Setaria italica*, or *Sorghum bicolor* were conducted with TBtools (v1.120). Orthologous gene pairs were subsequently analyzed using PAL2NAL (http://www.bork.embl.de/pal2nal/, accessed on 15 September 2022), which was used for the calculation of the synonymous and non-synonymous substitution rates (Ks and Ka, respectively).

### 4.5. RNA-Seq and Weighted Gene Co-Expression Network Analyses

The RNA-seq data of the 127 identified *ZmWRKY* genes in various tissues, including maize kernels collected at 14 days after pollination (DAP), endosperm samples collected at 27 DAP, embryo samples collected at 27 DAP, and root, silk, ear, internode, leaf, tassel stem, and tassel samples, were downloaded from the SRA database (accession number: SRX108496; http://www.ncbi.nlm.nih.gov/sra, accessed on 12 January 2023). Reads were trimmed for quality control with Trimmomatic v0.38 [61], and HISAT2 v2.0.05 was used to map the resultant cleaned reads to the B73 maize reference genome (RefGen_v5) [62]. FPKM (Fragments per kilobase of exon per million mapped reads) values for each gene were computed with Stringtie v2.2.0 based on comparisons of mean gene expression levels. Representative *ZmWRKY* genes from different groups were then selected and used to construct a gene expression heatmap in TBtools (v1.120).

Expression levels for identified *ZmWRKY* genes were assessed at 3, 6, and 12 hpi with *U. maydis* (Inf samples), while ddH_2_O-treated samples served as controls (CK) [23]. Transcriptomic data were assessed based on FPKM values as reported previously by Trapnell et al. [63], and these data were used to conduct a weighted gene co-expression network analysis (WGCNA) using R 3.6.0 to identify those gene modules exhibiting distinct expression patterns between the pooled infected and control samples [64]. Similar expression patterns between genes in these analyses were defined by a correlation coefficient threshold of r^2^ = 0.8, and the soft threshold parameter for the adjacency matrix was 12 (Figure S4). Gene expression was then analyzed after constructing a hierarchical clustering tree, and module–trait relationships were analyzed. *ZmWRKY* TF expression levels following *U. maydis* inoculation were computed in log_2_ (FPKM) values and presented in a heatmap using TBtools (v1.120).

### 4.6. qPCR Validation

Three biological replicate samples were collected for qPCR validation analyses. RNA was extracted from these samples with a FastPure^®^ Plant Total RNA Isolation Kit (Nanjing Vazyme Biotech Co. Ltd., Nanjing, China) based on provided directions, with RNA integrity, quality, and quantity then being assessed with NanoDrop 2000 (Thermo Fisher Scientific, Waltham, MA, USA), Agilent 4200 TapeStation (Agilent Technologies, Waldbron, Germany), and 1% agarose gel electrophoresis approaches. The FastKing gDNA Dispelling RT SuperMix (Tiangen Biotech Co. Ltd., Beijing, China) was then used for cDNA synthesis. Seven representative *ZmWRKY* genes were selected based on the FPKM values in the above transcriptomic analyses, and the expression of these genes was assessed via qPCR with a Bio-Rad CFX-96 PCR instrument (Bio-Rad Laboratories, Inc., Hercules, CA, USA). Thermocycler settings were as follows: 95 °C for 15 min, 40 cycles of 95 °C for 10 s, and 58 °C for 30 s. Relative gene expression levels were assessed with the 2^−∆∆Ct^ method [65], and *ZmUBI* served as a control gene for normalization. Primer v5.0 was used to design all primers in this study, which are listed in Table S12.

### 4.7. Yeast One-Hybrid Assay

Protein–DNA interactions between seven representative ZmWRKYs and the *ZmSWEET4b* promoter were evaluated through a Y1H assay. Briefly, the full-length coding sequences for these *ZmWRKYs* were inserted into the pB42AD vector using the *EcoR I* site, while the 2000 bp, 1 to 522 bp (P1), and 529 to 2000 bp (P2) promoter regions upstream of the *ZmSWEET4b* transcriptional start site were inserted into the pLacZi reporter plasmid using the *Xho I* site with an In-Fusion Cloning kit (Takara Biotech Co., Ltd., Osaka, Japan) (Table S13). The LacZ system was then used for Y1H screening. Briefly, the EGY47 yeast strain was transformed with these recombinant vectors or the control empty pB42AD vector. Transformants were spread onto the SD/-Trp/-Ura agar medium followed by incubation for 3 days at 28 °C. Positive colonies were then spread onto the SD/-Trp/-Ura/-Gal/-Raf/X-Gal agar medium and cultured at 28 °C.

### 4.8. Subcellular Localization Analyses

ZmWRKY protein subcellular localization predictions were made with WolfPsort WoLF PSORT (https://wolfpsort.hgc.jp/, accessed on 12 January 2023) and Plant-mPLoc (http://www.csbio.sjtu.edu.cn/bioinf/plant-multi/, accessed on 5 March 2023). The full-length coding sequences of appropriate ZmWRKY proteins were amplified and inserted into the pCambia1305-GFP vector downstream of the N-terminus of GFP using the *XbaI* site to generate ZmWRKY001/017/042/071/078/080/093-GFP fusion proteins regulated by the CaMV 35S promoter (Table S13). The resultant vectors were then used to transform *N. benthamiana*. Subsequent imaging was performed with a confocal microscope (Zeiss LSM900, Jena, Germany).

## Data Availability

All data analyzed during this study are provided in this published article and Supplementary Materials.

## Supplementary Materials

The following supporting information can be downloaded at: https://www.mdpi.com/article/10.3390/ijms241914916/s1, Figure S1. Collinearity analysis of WRKY genes between maize and 4 other plants. Figure S2. qPCR analyses of ZmWRKY genes from the *ZmWRKY060* in clade I, *ZmWRKY038*, *ZmWRKY082*, *ZmWRKY115* in clade II, *ZmWRKY013*, *ZmWRKY037*, *ZmWRKY114* in clade III; *ZmWRKY006*, *ZmWRKY021*, *ZmWRKY022* in clade V; *ZmWRKY028*, *ZmWRKY083 ZmWRKY117* in clade VI, respectively. Figure S3. Yeast one-hybrid assay results for analyses of interactions between ZmWRKY proteins and the *ZmSWEET4b* promoter segmentation. Figure S4. Soft threshold selection. Table S1. Number of transcription factors. Table S2. Basic information of *WRKY* genes in Maize. Table S3. Analyses the motifs in maize WRKY proteins from the MEME website. Table S4. Ka/Ks anaylsis of the duplicated *WRKY* gene pairs in *Zea mays*. Table S5. The Ka/Ks ratios for WRKY orthologous proteins between *Zea mays* and *Arabidopsis thaliana*. Table S6. The Ka/Ks ratios for WRKY orthologous proteins between *Zea mays* and *Oryza sativa*. Table S7. The Ka/Ks ratios for WRKY orthologous proteins between *Zea mays* and *Setaria italica*. Table S8. The Ka/Ks ratios for WRKY orthologous proteins between *Zea mays* and *Sorghum bicolor*. Table S9. Expression profiles of *ZmWRKY* genes in multiple tissues. Table S10. Expression profiles of *ZmWRKY* genes during the *U. maydis* infection. Table S11. W-box Sequence. Table S12. qPCR primer. Table S13. Vector construction primer.
