# Supplementary material for: Genome-Wide Characterization of the Maize (Zea mays L.) WRKY Transcription Factor Family and Their Responses to Ustilago maydis
Source: Int J Mol Sci. 2023 Oct 5;24(19):14916. doi: 10.3390/ijms241914916 (PMC10573107; doi:10.3390/ijms241914916)
Supplement: Supplementary file 1 [file ijms-24-14916-s001.zip › Supplementary Figure.pdf]

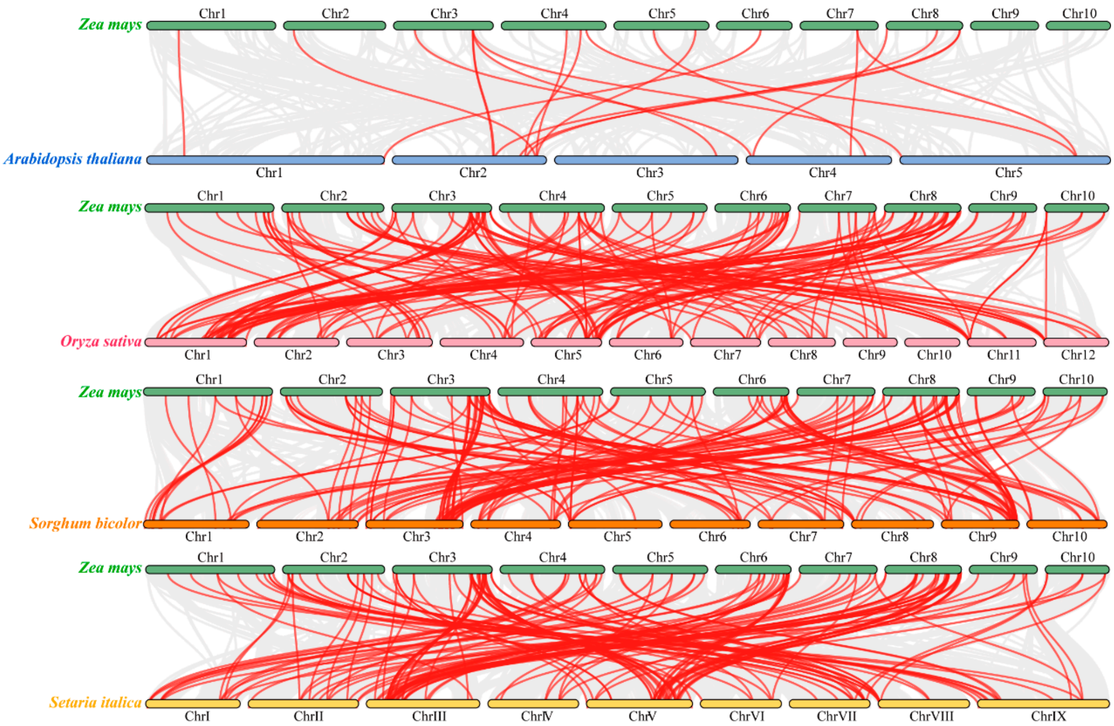

Figure S1. Collinearity analysis of WRKY genes between maize and 4 other plants. Grey line indicates the collinearity genes within maize and other plant genomes, the red curve indicates syntenic WRKY genes.

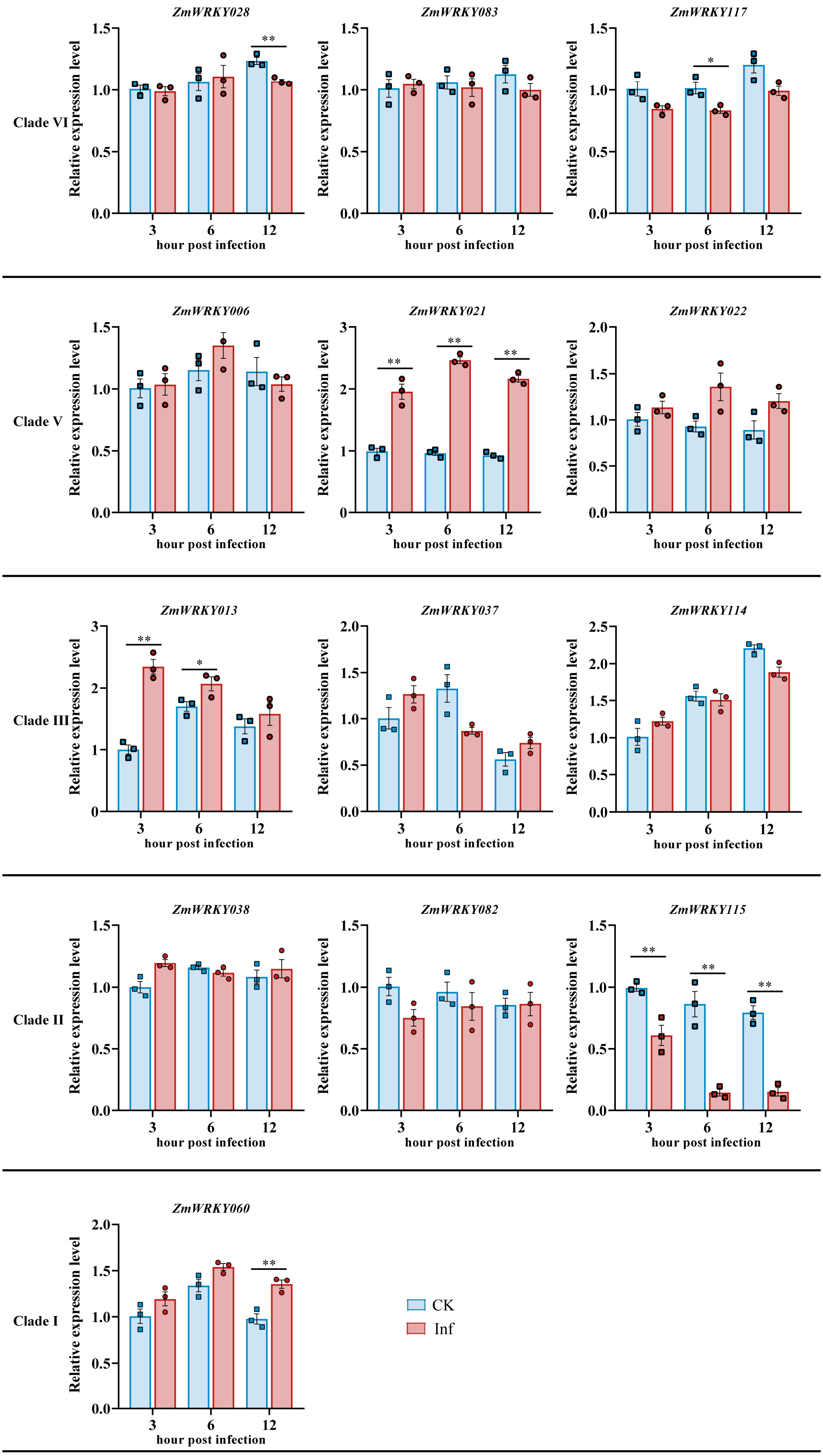

Clade VI
ZmWRKY028
ZmWRKY083
ZmWRKY117
Clade V
ZmWRKY006
ZmWRKY021
ZmWRKY022
Clade III
ZmWRKY013
ZmWRKY037
ZmWRKY114
Clade II
ZmWRKY038
ZmWRKY082
ZmWRKY115
Clade I
ZmWRKY060
Relative expression level
hour post infection
CK
Inf

Figure S2. qPCR analyses of *ZmWRKY* genes from the *ZmWRKY060* in clade I, *ZmWRKY038*, *ZmWRKY 082*, *ZmWRKY115* in clade II, *ZmWRKY013*, *ZmWRKY037*, *ZmWRKY114* in clade III; *ZmWRKY006*, *ZmWRKY021*, *ZmWRKY022* in clade V; *ZmWRKY028*, *ZmWRKY083 ZmWRKY117* in clade VI, respectively. Data are means ± standard errors from three technical and biological replicates. *P < 0.05, **P < 0.01; Student's t-test.

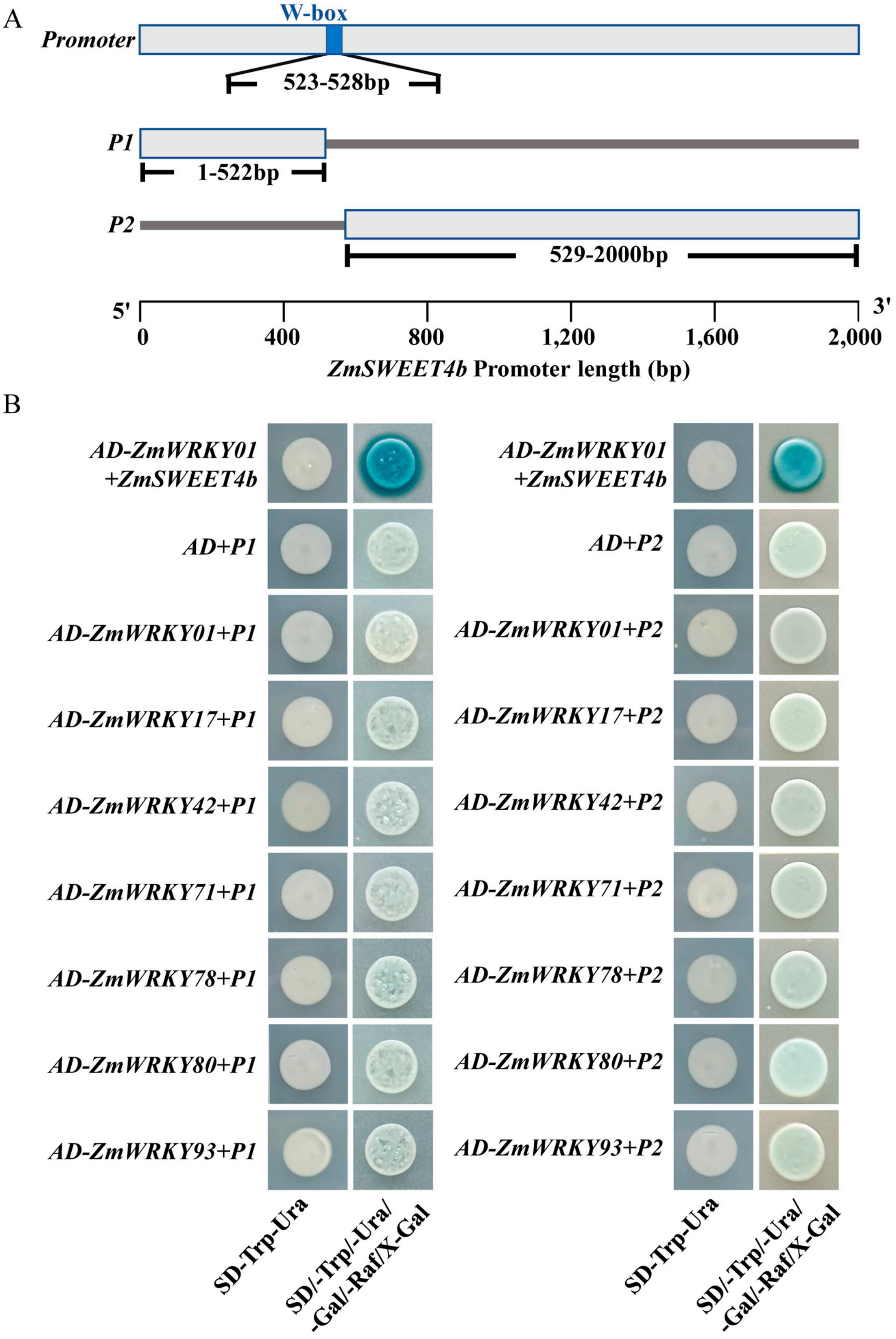

Figure S3. Yeast one-hybrid assay results for analyses of interactions between ZmWRKY proteins and the *ZmSWEET4b* promoter segmentation. (A) Model overview of *ZmSWEET4b* promoter segmentation. (B) Yeast one-hybrid assays between the *ZmSWEET4b* promoter segmentation and seven ZmWRKY proteins.

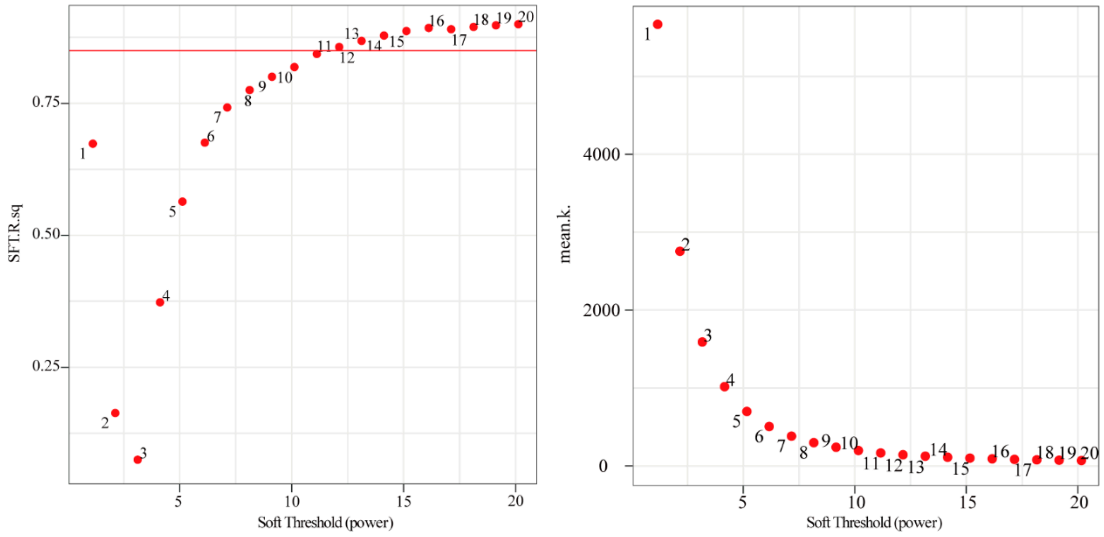

Figure S4. Soft threshold selection.
